# An ethnobotanical study of medicinal plants in Wonago Woreda, SNNPR, Ethiopia

**DOI:** 10.1186/1746-4269-5-28

**Published:** 2009-10-12

**Authors:** Fisseha Mesfin, Sebsebe Demissew, Tilahun Teklehaymanot

**Affiliations:** 1 Biology Department, Addis Ababa University, PO Box 1176, Addis Ababa, Ethiopia; 2 Aklilu Lemma Institute of Pathobiology, PO Box 56478, Addis Ababa University, Ethiopia

## Abstract

**Background:**

Medicinal plants are the integral part of the variety of cultures in Ethiopia and have been used over many centuries. Hence, the aim of this study is to document the medicinal plants in the natural vegetation and home gardens in Wonago Woreda, Gedeo Zone, Southern Nations, Nationalities and Peoples Regional State (SNNPR).

**Materials and methods:**

Thirty healers were selected to collect data on management of medicinal plants using semi-structured interview, group discussion, and field observation. The distribution of plant species in the study areas was surveyed, and preference ranking, direct matrix ranking, priority ranking of factors and Informant consensus factor (ICF) were calculated.

**Results:**

The informants categorized the vegetation into five community types based on plant density and associated landform: 'Raqqa', 'Hakka cadanaba', 'Mancchha', 'Bullukko', and 'Wodae gido'. 155 plant species were collected from the natural vegetation and 65 plant species from the home gardens ('Gattae Oduma'). Seventy-two plant species were documented as having medicinal value: Sixty-five (71%) from natural vegetation and 27 (29%) from home gardens. Forty-five (62%) were used for humans, 15(21%) for livestock and 13(18%) for treating both human and livestock ailments: 35 (43.2%) were Shrubs, 28(34.5%) herbs, 17 (20.9%) trees and 1(1.2%) climbers. The root (35.8%) was the most commonly used plant part. The category: malaria, fever and headache had the highest 0.82 ICF. Agricultural expansion (24.4%) in the area was found to be the main threat for medicinal plants followed by fire wood collection (18.8%). Peoples' culture and spiritual beliefs somehow helped in the conservation of medicinal plants.

**Conclusion:**

Traditional healers still depend largely on naturally growing plant species and the important medicinal plants are under threat. The documented medicinal plants can serve as a basis for further studies on the regions medicinal plants knowledge and for future phytochemical and pharmacological studies.

## Introduction

Ethiopians have used traditional medicines for many centuries, the use of which has become an integral part of the different cultures in Ethiopia. The indigenous peoples of different localities in the country have developed their own specific knowledge of plant resource uses, management and conservation [1].

Traditional remedies are sometimes the only source of therapeutics for nearly 80% of human population and 90% of livestock in Ethiopia of which 95% are plant origin [2]. The majority of the population that lives in the rural and the poor people in urban areas rely mainly on traditional medicines to meet their primary health care needs.

In most scenarios, the traditional knowledge in Ethiopia is passed verbally from generation to generation and valuable information can be lost whenever a traditional medical practitioner passes without conveying his traditional medicinal plants knowledge. In addition, the loss of valuable medicinal plants due to population pressure, agricultural expansion and deforestation is widely reported by different workers [3,4]. As a result, the need to perform ethnobotanical researches and to document the medicinal plants and the associated indigenous knowledge must be an urgent task [5,6].

The studies conducted on the traditional medicinal plants in Ethiopia are limited when compared with the multiethnic cultural diversity and the diverse flora of Ethiopia. Thus, this study was initiated to document the medicinal plants in the natural vegetation and home gardens in Wonago Woreda, which assume that the data could be used as a basis for further studies on medicinal plants in Wonago Woreda and for future phytochemical and pharmacological studies.

## Materials and methods

### Study sites

**Figure 1:**
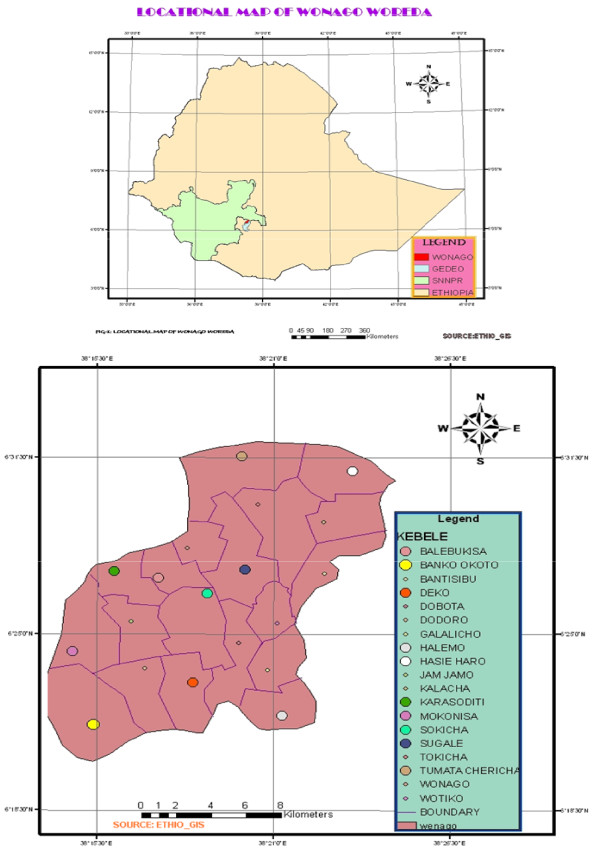
**Location of Wonago Woreda in Gedeo zone; Southern Nations, Nationalities and Peoples Regional State (SNNPR)**.

Wonago Woreda (N 6° 20' and E 38° 19') is located 380 km from Addis Ababa in Gedeo Zone, Southern Nations, Nationalities and Peoples Regional State (SNNPR) and bordering with Oromia to the west and northwest, Yirgachefee to the south and southeast, Dilla to the north and Bule to the east. It is approximately 248 sq. km (24,790 ha) and comprises of 19 Kebeles (Fig. 1).

The 2005 census indicates that Wonago Woreda has a total population of 162,663 of which 78,649 (48.3%) are males and 84,014 (51.6%) are females. The population density of the Woreda is 702 persons per km^2 ^at a national growth rate of 1.07 percent. Seventy four percent of the population in the Woreda are the Gedeo people.

**Figure 2:**
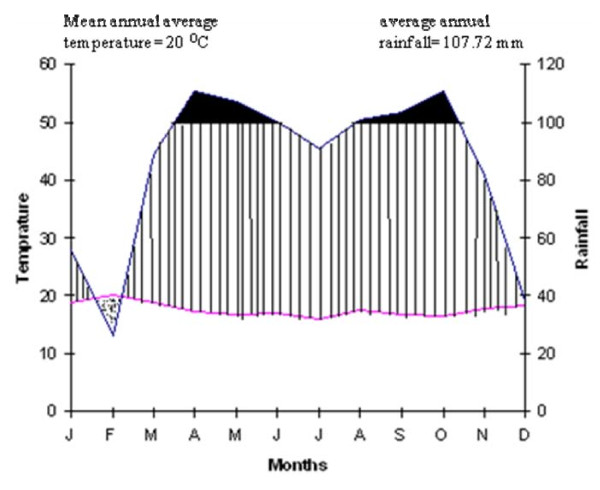
**Climatogram of the study area from 1996 to 2005 at Kotty Weather Station, Wonago Woreda in Gedeo zone**. Source: National Meteorological Service Agency.

As the agricultural sector is the dominant means of livelihood for the majority of Wonago Woreda people, out of the total of 24,790 hectares of land in the Woreda, 22,871 hectares are known to have potential for agriculture. Annual crops cover 5.03 percent; perennial crops 84.77 percent, uncultivable land 0.65 percent and others are 3.52 percent. It has three main agro-climatic zones with the topography ranging from wide flat valley bottoms to steep mountain slopes. The rainfall distribution of the study area is bimodal. The main rainy season is from June to September ('Kiremt' or Mahar') and the short rainy season is from February to April ('Belg'). The average annual rainfall is 107.72 mm and, the mean annual average temperature of the Woreda is 20°C (Fig. 2)

The study was conducted in ten kebeles (farmers' associations) in Wonago Woreda, SNNPR from November 1, 2006 to December 3, 2006. Prior to ethnobotanical data collection, discussions were made with elders and local authorities to select the kebeles where traditional healers were found. The kebeles were selected based on availability of traditional healers, and on the recommendations of elders and local authorities in the Wonago Woreda: 'Bankookoto', 'Balebukisa', 'Deko', 'Halemo', 'Haseharo', 'Karasodity', 'Mokonisa', 'Sokicha', 'Sugale', and 'Tumata cherecha'(Fig. 1).

### Ethnobotanical data collection

Thirty traditional healers (22 males and 8 females) were selected from Gedeo people in the Wonago Woreda based on the recommendation from elders and local authorities (Development Agents and Kebele administration leaders). The ages of the healers were between 35 years and 75 years. A brief group discussion was made with the informants at each kebele prior to ethnobotanical data collection to get their consent and to explain to them that their cooperation is a valuable contribution to the documentation of the traditional medicinal plants of the Wonago Woreda. Semi-structured interview, group discussion, and field observation were employed to collect data on knowledge and management of medicinal plants [7-9]. The group discussions were conducted to elaborate the methods of preparation, administration and conservation of the medicinal plants. Interviews were conducted in "Gedeoffa" language with the help of local translator. During the study period, each informant was visited two to three times in order to confirm the reliability of the ethnobotanical information. The responses that were not in harmony with each other were rejected.

### Plant specimens' collections and identifications

The reported medicinal plants were collected from natural vegetation and home gardens during the field walks and trees, shrubs, herbs and climbers were listed. Voucher specimens were collected, pressed and deposited in the National Herbarium of Addis Ababa University (AAU). The plants identification was performed both in the field, and at the National Herbarium of AAU [10-16].

### Data analysis

A descriptive statistical methods, percentage and frequency were used to analyze the ethnobotanical data on reported medicinal plants and associated indigenious knowledge.

Preference ranking was computed to assess the degree of effectiveness of certain medicinal plants against most prevalent diseases in the area. Priority ranking of factors perceived as threats to medicinal plants based on their level of destructive effects (values 1-6 were given: 1 is the least destructive threat, and 6 is the most destructive threat) and Direct matrix ranking on uses perceived as threats to medicinal plants were conducted for multipurpose medicinal plants that were commonly reported by healers [7,9].

The Informant consensus factor (ICF) was calculated for each category to identify the agreements of the informants on the reported cures for the group of diseases. The ICF was calculated as follows: number of use citations in each category (n_ur_) minus the number of species used (n_t_), and divided by the numbers of use citations in each category minus one [17].



## Results

### Local categories of vegetation

The local communities categorized the vegetation of the study area into five types based on plant density and associated landform.

I. **'**Raqqa' refers to densely forested land. Currently, this type of vegetation has declined in the study area because of degradation by human activities, over grazing, and climate changes.

II. 'Hakka Cadanaba' refers to vegetation growing in marshy or water logged areas often characterized by salty soil. Plant species such as *Phoenix reclinata *and *Cyperus spp*. were more frequent.

III. 'Mancchha' refers to a bare or with poor vegetation with some types of herbs and grasses appearing only during the rainy season.

IV. 'Bullukko' refers to the heterogeneous mixture of shrubs and grass communities not suitable for agriculture.

V. 'Wodae Gido' refers to wooded and under-growing herbaceous vegetation growing along riversides. Plant species like *Spatodea nilotica, Erythrina brucei, Ficus spp*. and *Arundo donax *were common.

### Plant species in the natural vegetation of the study area

155 plant species were collected from the natural vegetation, which were distributed among 63 families and 136 genera. The leading family was Asteraceae with 18 species, followed by Fabaceae with 12 species, Euphorbiaceae with 9 species, Poaceae, Solanaceae and Rosaceae each with 6 species and Myrtaceae with 5 species. Fifty-seven (37%) were herbs, 53 (34%) were shrubs, 39 (25%) were trees, 5 (3%) were climbers, and one (1%) was epiphyte [see Additional file 1].

Forty-two percent of 155 plant species were medicinal plants. They were distributed among 39 families and 63 genera. The leading family was Asteraceae with 7 species, followed by Euphorbiaceae with 6 species, Fabaceae with 5 species, Solanaceae with 4 species: 31 (49%) were shrubs, 17(27%) were herbs, and 15 (24%) were trees.

### Plant diversity of the 'Gattae Oduma' (Home garden)

In the 'Gattae Oduma' (Home garden), the farmers grew diverse plant species with known uses. The number of plants recorded represents 65 species that belong to 33 families and 57 genera. In terms of species composition, Solanaceae had 6 species followed by Poaceae with 5 species, Asteraceae, Fabaceae, Lamiaceae and Rosaceae each with 4 species and Brassicaceae, Euphorbiaceae and Rutaceae each with 3 species (Table 1).

**Table 1:** List of plant species in home garden in the study area, Wonago Woreda (Habit: T-tree, Sh-shrub, H-herb, and Cl-climber. Uses: Sp-spice, F-food, M-medicine, CI- cash income, Fn-fence, Or-ornamental, and St-stimulant)

**Family**	**Plant species**	**Local name**	**Habit**	**Use**	**Voucher No**.
Acanthaceae	*Justicia schimperiana *(Hochst.ex Nees) T. Anders	Dhumuga	S	M, Fn	FM30
Alliaceae	*Allium cepa *L.	Kagelcha Sunkurtae	H	F	FM14
Alliaceae	*Allium sativum *L.	Dimoxxa sunkurtae	H	F, M	FM15
Anacardiaceae	*Mangifera indica *L.	Mango	T	F, CI	FM61
Anacardiaceae	*Rhus vulgaris *Meikle	Suggutae	Sh	M	FM57
Annonaceae	*Annona squamosa *L.	Gishta	S	F	FM18
Apiaceae	*Daucus carota *L.	Karoti	H	F	FM36
Araceae	*Colocasia esculenta *(L.) Schott	Godarre	H	F, M	FM43
Arecaceae	*Phoenix reclinata *Jacq.	Maxxaae	T	Or	FM66
Asteraceae	*Artemisia abyssinica *Sch.Bip. ex A. Rich.	Sugetieae	H	M	FM17
Asteraceae	*Artemisia afra *Jack. ex Wild	Chugughee	H	M	FM38
Asteraceae	*Helianthus annuus *L.	Suufii	H	F, M	FM65
Asteraceae	*Vernonia amygdalina *Del.	Ebicha	S	M	FM31
Brassicaceae	*Brassica carinata *A. Br.	Shaanna	H	F	FM23
Brassicaceae	*Brassica oleracea *L.	Faragae shaanna	H	F	FM70
Brassicaceae	*Lepidium sativum L*.	Faxxoo	H	M	FM20
Bromelianceae	*Ananas comosus L*.	Annanassae	H	F	FM45
Caricaceae	*Carica papaya *L.	Papaya	T	F, M	FM46
Celastraceae	*Catha edulis *(Vahl) Forssk. ex Endl.	Chatae	S	M, CI	FM19
Celastraceae	*Maytenus senegalensis *(Lam.) Excell	Shekko	Sh	M	FM54
Chenopodaceae	*Beta vulgaris L*.	Dammooxxa	H	F	FM24
Convolvulaceae	*Ipomeoea batatas *L.	Boynnaae	C	F,	FM41
Cucurbitaceae	*Cucurbita pepo *L.	Buqe	Cl	F, M	FM16
Dioscoreaceae	*Dioscorea praehensilis *Benth.	Qoco	Cl	F	FM28
Dracaenaceae	*Dracaena steudneri *Engl.	Afarfartu	T	M, Or	FM37
Euphorbiaceae	*Euphorbia candelabrum *Kostshy	Addama	Sh	Fn	FM48
Euphorbiaceae	*Euphorbia pulcherrima *(R. Grah.) Willd.	Ababa	S	Or	FM40
Euphorbiaceae	*Ricinus communis *L.	Qobo	S	Sp, CI	FM71
Fabaceae	*Cajanus cajan L*.	Atarra	H	F	FM44
Fabaceae	*Glycine max (*L.) Merr.	Atara	S	F	FM55
Fabaceae	*Phaseolus lunatus *L.	Coma	Cl	F	FM34
Fabaceae	*Vicia faba *L.	Baqqalleo	H	F	FM59
Flacourtiaceae	*Dovyalis abyssinica *(A. Rich.) Warb	Akuku	S	Fn, Or	FM13
Lamiaceae	*Ocimum basilicum *L.	Basobila	H	F	FM67
Lamiaceae	*Ocimum lamiifolium *Benth.	Damakase	H	M	FM52
Lamiaceae	*Otostegia tomentosa *A.Rich	Tunjuti	S	Fn	FM63
Lamiaceae	*Plectranthus edulis *Vatke	Dinich-Oromo	H	F	FM60
Lauraceae	*Persea americana *Mill.	Abokado	T	F, CI	FM75
Malvaceae	*Gossypium herbaceum *L.	Jirbi	S	M, CI	FM29
Moringaceae	*Moringa stenopetala L*.	Shifferaw	T	M, Or	FM62
Musaceae	*Ensete ventricosum *(Welw.) Cheesman	Warqo	Sh	M, O	FM5
Musaceae	*Musa paradisiaca *L.	Musi	H	F, Or	FM33
Poaceae	*Eragrostis tef *(Zucc.) Trotter	Xxaffae	H	F	FM22
Poaceae	*Hordeum vulgare L*.	Dinnaae	H	F	FM21
Poaceae	*Saccharum officinarum *L.	Shunkora	H	F, CI	FM72
Poaceae	*Sorghum vulgare *Pers.	Agadae	H	F	FM35
Poaceae	*Zea mays *L.	Beedeella	H	F, CI	FM58
Punicaceae	*Punica granatum *L.	Romanoo	S	F	FM68
Rhamnaceae	*Rhamnus prinoides *L'Herit.	Geshae	S	CI	FM47
Rosaceae	*Malus sylvestris *Mill		T	F	FM53
Rosaceae	*Prunus persica *(L.) Batsch	Kokae	S	F	FM32
Rosaceae	*Rosa abyssinica *Lindley	Xigeradao	Sh	Or	FM6
Rosaceae	*Rubus steudneri *Shweinf.	Engorrei	Sh	F, Or	FM74
Rubiaceae	*Coffea arabica *L.	Buno	S	M, CI	FM1
Rutaceae	*Citrus limon *(L.) Burm.f.	Lomae	S	F, M	FM64
Rutaceae	*Citrus medica *L.	Trungo	S	F	FM27
Rutaceae	*Ruta chalepensis *L.	Ciladami	H	M	FM50
Solanaceae	*Capsicum annum *L.	Miximixo	H	F, M	FM25
Solanaceae	*Capsicum frutescens L*.	Bereberae	H	F	FM26
Solanaceae	*Datura stramonium L*.	Atsefareceae	H	M	FM47
Solanaceae	*Lycopersicon esculentum *Mill	Timatimi	H	F	FM42
Solanaceae	*Nicotiana tabacum *L.	Tambo	H	CI, M	FM56
Solanaceae	*Solanum americanum *Miller	Dinicha	Sh	F	FM73
Zingebraceae	*Aframomum corrorima *(Braun) Jansen.	Okkoshae	H	Sp	FM39
Zingiberaceae	*Zingiber officinale *Roscoe	Jaanjiibeello	H	F, M	FM51

Out of the Sixty-five 'Gattae Oduma' plant species, 31(48%) were herbs, 23(35%) were shrubs, 7 (11%) were trees and 4 (6%) were climbers. The home gardens' flora were composed of 25 (38%) food, 10(15%) medicinal and 30(46%) other useful plant species. Majority of the plant species in the home gardens (48%) provided at least two of the uses listed in Table 2.

**Table 2:** Service categories of home garden plants ('Gattae Oduma') in the study area, Wonago Woreda

**Service categories**	**No. species**	**% of the total species**
Cash income	1	2%
Cash income, Stimulant	1	2%
Fence	2	3%
Fence and Ornament	1	2%
Food	25	38%
Food and Cash income	4	6%
Food and Medicine	8	12%
Food and Ornament	2	3%
Medicine	10	15%
Medicine and Cash income	3	5%
Medicine and Fence	1	2%
Medicine and Ornament	3	5%
Ornament	3	5%
Spice	1	2%
Spice and Cash income	1	2%

### Medicinal plants

#### Medicinal plants used to treat human and livestock diseases

The highest medicinal plant knowledge acquisition by the healers in this study site was from parents or close relatives (91%) followed by self trial and error method (9%). The healers have a very high intention to keep their traditional knowledge secrete and less than 2% of them were ready to transfer their knowledge on incentive bases.

Seventy-two plant species distributed into 48 families and 70 genera were documented as having medicinal value in the study area. Sixty-five (71%) of the medicinal plants were collected from natural vegetation and 27 (29%) from home gardens. Of these 45(62%) were used as human medicines (Table 3), 15(21%) as livestock medicines (Table 4) and 13(18%) were used for treating both human and livestock diseases (Table 5).

**Table 3:** List of medicinal plants for treating human diseases in the study area, Wonago Woreda

**Families**	**Scientific name**	**Local name**	**Habit**	**Preparation and application**	**Diseases treated**	**Voucher Number**
Acanthaceae	*Justicia schimperiana *(Hochst.ex A. Nees) T.Anders	Dummiuggae	Sh	Pounded fresh/dry leaves is concocted with bark of *Croton macrostachyus *is taken orally for three days.	Intestinal parasites	FM30
Alliaceae	*Allium sativum *L.	'Sunkurtae'	H	Fresh or dry fruits is Chewed and orally	Malaria	FM15
Apiaceae	*Foeniculum vulgare *Mill	Melloo	H	Pounded dry/fresh root is taken with coffee or tea as drink.	Abdominal pain	FM193
Araceae	*Colocasia esculenta *(L.) Schott.	Godarre	H	Crushed/pounded dry/fresh concocted with *Zingiber officinale *rhizome is taken with coffee as drink.	Diarrhea	FM43
				Fine powder of plant part mixed with water and mixture drunk or thick paste applied to affected part	Trachoma	
Asclepidaceae	*Gomphocarpus purpurascens *A. Rich	Mexxino	Sh	Pound fresh/dry root bark with water is taken as a drink	Febrile illness	FM142
Asclepidaceae	*Kanahala laniflora *(Forssk.) R. Br.	Wundiffo	Sh	Pounded fresh/dry root concocted with roots of *Croton macrostachys *and *Senna occidentalis *is taken orally	Amoebas	FM136
				Pounded fresh/dry root concocted with roots of *Croton macrostachys *and *Senna occidentalis *and mixed with butter is taken orally	Bronchitis	
				Fresh/dry root powder mixed with honey is taken orally before breakfast for three days.	Hepatitis	
Asparagaceae	*Asparagus africanus *L.	'Uffae '	Sh	Powder of dry root with butter is applied on wound	Wound	FM206
Asteraceae	*Artemisia abyssinica *Sch.Bip. ex A. Rich	Sugetieae	H	Crushed or pounded fresh stem with butter is applied topically	Eye infection	FM17
Asteraceae	*Artemisia afra *Jack. ex Wild	Chugughee	H	Crushed or pounded fresh or dry leaves are boiled in water and the filtrate is taken hot; orally	Abdominal pain	FM38
				Fresh leaves are chewed and taken orally	Headache	
				Powdered fresh/dry leaves nixed with butter is taken with coffee orally before breakfast for three days	Malaria	
Asteraceae	*Carduus leptacanthus *Fresen.	Guccino	H	Powdered dry stem mixed with butter is taken with coffee or tea.	Ascariasis	FM86
				Crushed/pounded dry stem concocted with *Vernonia amygdalina *leaves mixed with water is taken orally	Haemorrhoid	
Asteraceae	*Helianthus annuus *L.	Suffae	H	Mix the powder with water and drink	Food poison	FM65
Asteraceae	*Vernonia amygdalina *Del.	Ebicha	Sh	Crushed, pounded and mix with little water then drink for five days.	Diarrhea	FM31
				Wash the patient body with the plant part and drink for three days.		
Asteraceae	*Vernonia auriculifera *Hiern	Dangireto	Sh	Crushed, pounded and mix with cold water, one cup of the filtrate is given for adult, one-half of the cup for children for three days	Snake poison	FM144
Asteraceae	*Xantium strumarium *L.	Dehanekayae	H	The plant part squeezing it through clean locally made cloth for five days on affected part or wash the affected part for both diseases.	Skin infection	FM9
Boraginaceae	Cynoglossum lanceolatum Forsk.	Korchibae	H	Handful root is crushed by hand, small amount of cold water is added to squash, the mixture is inhaled and few drops are drunk.	Fertility & abnormal growth	FM114
				Crushed, pounded and mix with water and drink.	Mental problems	
Boragnaceae	*Cordia africana *Lam.	Waddissa	T	Powdered dry root bark is sprinkled on burning charcoal and smoke is inhaled covered by cloth	Evil eye	FM167
Brassicaceae	*Lepidium sativum *L.	Feaxxo	H	Dry seed powder is taken as with coffee as drink	Intestinal parasites	FM20
				Pounded seeds mixed with *Allium sativum *bulbs and honey is taken orally for five days before breakfast After each dose, one glass of melted butter is recommended for immediate recovery.	Malaria	
				Dry seed powder with pounded seed of *Ocimum lamiifolium *is taken with coffee as drink	'Mich'	
				Dry seed powder with pounded seed of *Ocimum lamiifolium *is taken with coffee as drink	Headache	
Caricaceae	*Carica papaya *L.	Papaya	T	Chewed and swallowed fresh seed	Amoebas	FM46
				Chew and swallow seed	Intestinal parasite	
Caryophyllaceae	*Stellaria sennii *Chiov.		H	Decoction root	Hepatitis	FM188
Celastraceae	*Catha edulis *(Vahl.) Forssk ex Endl.	Chatae	Sh	Crushed/pounded fresh stem concocted with leaves of *Vernonia amygdalina *is boiled and one glass of the filtrate is taken orally	Urine retention	FM19
Celastraceae	*Maytenus senegalensis *(Lam.) Excell	Shekko	Sh	Powdered fresh/dry seed with water or butter is taken with coffee or tea as drink for five days.	Epilepsy	FM54
				Powdered fresh/dry seed with *Ocimum lamiifolium *seed is take with coffee as drink	Headache	
Cucurbitaceae	*Lagenaria siceraria *(Molina) Standl.	Botto	H	Ripe fruits including seeds are immersed in water for overnight; the water is taken orally in the morning before breakfast.	Gonorrhea	FM205
Cucurbitaceae	*Momordica foetida *Schumach	Yubarrae	Sh	Crushed/pounded fresh/dry root mixed with *Allium sativum *bulb is taken orally before breakfast for three days.	Bronchitis	FM108
				Infusion of fresh/dry root powder is taken orally	Food poison	
Dracaenaceae	*Dracaena steudneri *Engl.	Afrafartu	T	Powder of dry root is applied to wound.	Wound	FM37
Euphorbiaceae	*Croton macrostachyus *Del.	Bissano	T	Crushed/pounded fresh/dry leaves boiled with water is concocted with *Allium sativum *(bulb) roasted with butter and left over night outside home is taken orally at the morning	Malaria	FM162
				Rubbing affected part by exudates of old leaves	Ringworm	
Euphorbiaceae	*Euphorbia candelabrum *Kostshy	Addama	Sh	Milky latex from plant mixed with roots powder of *Ruta chalepensis *and paste applied to affected area	Ringworm	FM48
Euphorbiaceae	*Euphorbia tirucalli *L.	Kinchibae	Sh	Rubbing affected part with crushed fresh/dry root concocted with crushed leaves of *Coffea arabica*	'Kintarot'	FM40
Euphorbiaceae	*Ricinus communis *L.	Gulloo	Sh	Crushed/pounded leaves with coffee, tea or milk is taken as a drunk before copulation	impotency	FM71
Euphorbiaceae	*Tragia cinerea *(Pax) Gilbert & Radcl. Smith	Alebelabitae	H	Fine powder of plant part mixed with butter and drink before sexual intercourse with his partner.	'Kintarot'	FM87
				Fine powder of plant part mix with honey and drink before sexual intercourse		
Fabaceae	*Millettia ferruginea *(Hochst.) Bark	Berberae	T	Fresh/dry fruits powder with butter is applied topically	Skin infection	FM190
Fabaceae	*Senna occidentalis *(L.) Link	Assenmeka	H	fresh root powder mixed with water is taken as a drink for three days	Bleeding nose	FM103
				Fresh root powder with butter is taken as a drink for before breakfast three days.	Excessive menstruation	
				Fresh root powder with honey is taken as a drink for before copulation	Gonorrhea	
				Chewing and swallowing fresh root	Tonsillitis	
Lamiaceae	*Ocimum lamiifolium *Hochst. Ex Benth.	Damakase	H	Pounded fresh leaves mixed with butter is taken with coffee as drink at the morning	Cough	FM52
Lognaceae	*Buddleja polystachya *Fresen	Affarao	Sh	Infusion of crushed/pounded dry leaves is taken orally	'Dingetegia'	FM7
Malvaceae	*Gossypium arboretum *L.	Jirbiae	Sh	Powdered dry root bark infusion is taken as drunk	Lymphatic swelling	FM29
Malvaceae	*Sida schimperiana *Hochst. ex A.Rich	Gebresede	Sh	Crushed, pounded, and boiled with water and cooled for 2 hours and 2 glasses are served as a drink.	Epilepsy	FM170
Meliaceae	*Trichilia dregeana *Sond.	Yumbarro	T	Concoction root bark	Mental problems	FM126
Meliantaceae	*Bersama abyssinica *Fresen	Jejjebba	Sh	Crushed/pounded fresh root mixed with cold water is taken orally	Bronchitis	FM163
				Crushed/pounded fresh root concocted with leaves of *Ruta chalepensis *with water is taken orally	Febrile illness	
Moraceae	*Ficus ovata *Vahl	Shollae	T	powder of dry fruits mixed with butter is applied after scratching the affected area	Ringworm	FM153
Moringaceae	*Moringa stenopetala *L.	Sihferaw	T	Chewing and swallowing fresh leaves	Vomiting	FM62
Musaceae	*Ensete ventricosum *(Welw.) Cheesman	Warqo	Sh	Crushed/pounded fresh root with water is taken orally	Abdominal pain	FM5
				Crushed/pounded fresh root with water is taken orally	Amoebic dysentery	
Myrsinaceae	*Embelia schimperi *Vatke.	Sharrengo	Sh	Crushed fresh root with water is taken as a drink for several days	Leprosy	FM122
Myrtaceae	*Eucalyptus globules *Labill	D/barzafae	T	Inhalation of steam of young fresh leaves with stem before bedtime	'Mich'	FM150
Phytolaceae	*Phytolacca dodecandra *L'Herit	Indoodae	Sh	Pounded fresh/dry leaves mixed with water is taken orally before breakfast for three days.	Malaria	FM176
Podocarpaceae	*Podocarpus falcatus *(Thunb.) Mirb.	Zigbo	T	Fresh/dry root powder mixed with water is taken orally	Febrile illness	FM11
Polygonaceae	*Rumex nepalensis *Spreng.	Dangago	H	Paste of fresh/dry stem powder with butter is applied topically	Wound	FM10
Resedaceae	*Caylusea abyssinica *(Fresen.) Fish. & Mey.	Sheggitae	H	Crushed/pounded fresh/dry root water is taken orally	Ascariasis	FM131
Rosaceae	*Hagenia abyssinica *(Brucie.) J. F. Gmel	Kossae	T	Mix the powder with honey and a little bit of water and then boil and drink before breakfast for five days.	Ascariasis	FM120
				Mix the powder with local 'tella' and leave for overnight and drink before breakfast for three days		
Rosaceae	*Prunus africana *(Hook.F.) Kalkam	T/kaka	T	Crushed/pounded dry root bark mixed with water is taken as a drink	Ascariasis	FM209
				Dry root powder concocted with *Parthenium hysterophorus *root powder is taken orally for three days.	Gonorrhea	
Rubiaceae	*Coffea arabica *L.	Buno	Sh	Smoke inhalation of dried leaves and infusion of leaves is taken orally	Vomiting	FM1
Rubiaceae	*Pentas schimperiana *(A. Rich) Vatke	Dibexxo	Sh	Fresh/dry root bark powder mixed with water is taken orally	Epilepsy	FM78
Rutaceae	*Citrus limon *(L.)Burm.F.	Lomae	Sh	Chew and swallow fresh fruits	Cough	FM123
Rutaceae	*Ruta chalepensis *L.	Xenadamae	H	Crushed/pounded fresh leaves with water of or coffee is taken orally	'Dingetega'	FM50
				Chewing and swallowing fresh leaves	Stomach-ache	
				Chewing fresh leaves using the jaw with toothache	Toothache	
Sapindaceae	*Dodonaea angustifolia *L.F.	Ittechhae	Sh	Decoction of dry fruit is applied topically	Ectoparasite	FM83
				Powder dry fruits with water is taken orally.	Lymphatic swelling	
Simaroubaceae	*Brucea antidysenterica *J.F.Mill	Kapparro	Sh	Powdered fresh root bark mixed with water is applied topically	Wound	FM202
Solanaceae	*Capsicum annuum *L.	Miximixo	H	Chew and swallow fresh/dry fruits	Ascariasis	FM25
Tiliaceae	*Grewia ferruginea *Hochst ex A. Rich	Ogomdii	Sh	Crushed/pounded fresh/dry root bark concocted with root of *Ensete ventricosum *and mixed with water is kept over night and taken orally as a drink before breakfast.	Cough	FM121
				Pounded fresh/dry root bark mix with butter is taken as drink before breakfast for three days.	Evil eye	
Tiliaceae	*Triumfetta tomentosa *Boj.	Kombocho	Sh	Mix the powder with a little bit of local 'araqi' and then apply the paste to wound	Fire burn	FM171
Verbenaceae	*Lantana camara *L.	Yewef kollo	Sh	Dry stem powder mixed with water is taken orally	Diarrhea	FM146
Zingeberaceae	*Zingiber officinale Rosc*.	Jaanjiibeello	H	Chewed and swallowed	Stomach-ache	FM51

**Table 4:** List of medicinal plants for treating livestock diseases in the study area, Wonago Woreda

**Families**	**Scientific name**	**Local name**	**Habit**	**Preparation and application**	**Diseases treated**	**Voucher Number**
Acanthaceae	*Justicia schimperiana *(Hochst.ex A. Nees). Anders	Dummiuggae	Sh	Crushed, pounded fresh/dry leaf concocted with *Croton macrostachyus *in cold water is given as a drink for three days.	Intestinal parasites	FM30
Amaranthaceae	*Achyranthes aspera *L.	Derrgu	H	Powdered dry/fresh leaf with water is applied externally	Ectoparasite	FM115
				Powder of root mixed with water is given orally	Diarrhea	
Anacardiaceae	*Rhus vulgaris *Meikle	Suggutae	Sh	Crushed, pounded fresh/dry root mixed with cold water; kept outside for overnight is given as drink in the morning	Blackleg	FM57
Apocynaceae	*Maytenus arbutifolia *(A. Rich) Wilczek	Kombollechae	Sh	Powdered dry leaf mixed with butter is applied topically	Wound	FM138
Asparagaceae	*Asparagus africanus *L.	Uffae	Sh	Powder of dry root is applied topically	Wound	FM206
Asteraceae	*Cirsium englerianum *O. Hoffm.	Galigloo	H	Concoction of fresh/dry root mixed with residue of local 'tella' or 'areqie' is given as drink.	Urine with blood	FM64
				Crushed, pounded and mix with residue of local 'areqie' or 'tella' and drink.	Sterility	
				Powdered fresh leaf mixed with residue of local 'areqie' or 'tella' is given as drink	Anthrax	
				Crushed, pounded and mix with cold water, applied orally for three days	Snake poison	
				Fresh leaf is squeezed on to affected part for five days	Skin infection/Kintarot	
Asteraceae	*Vernonia auriculifera *Hiern	Dangireto	Sh	Crushed, pounded root mixed with cold water is administered orally	Snake poison	FM144
Asteraceae	*Xantium strumarium *L.	Dehanekayae	H	Squeezing leaf through clean locally made cloth for five days on affected part or wash the affected part	Wart, Skin infection	FM9
Boragnaceae	*Cordia africana *Lam.	Waddissa	T	Root bar is smoked in the barn	Evil eye	FM167
Casuarinaceae	*Casuarina cunninghamiana *Miq.	Shewshewae	T	Concoction of fresh/dry root bark mixed with leaf of *Croton macrostachyus *and water is given as drink.	Lymphatic swelling/Urine retention	FM76
Celastraceae	*Maytenus senegalensis *(Lam.) Excell	Shekko	Sh	Root powder mixed with leaf of *Ocimum lamiifolium *is administered orally	Febrile Disease	FM54
Clustiaceae	*Hypericum revolutum *Vahl		Sh	Leaf is pounded and mix with water applied orally.	Fattening	FM93
Cucurbitaceae	*Cucurbita pepo *L.	Buqe	Cl	Fresh/dry root ash mixed with butter is applied topically	Skin infection	FM16
Fabaceae	*Calpurnia aurea *(Alt.) Benth.	Chekketa	Sh	Seed powder mixed with butter is applied on infected eye.	Eye infection	FM98
				Powdered fresh/dry root with water is given orally.	Urine retention	
				Powdered fresh/dry root with butter is given orally	Black leg	
				Crushed, pounded fresh root with fresh leaf of *Vernonia amygdalina *mixed with residue of local areqie or tella is given orally	Anthrax	
				Crushed, pounded fresh root with fresh leaf of *Parthneium hysterophrus *mixed with residue of local areqie or tella is given orally	Blackleg	
Malvaceae	*Sida schimperiana *Hochst. ex A.Rich	Gebresede	Sh	Leaf powder is mixed with water is administered orally for three days before grazing	Mental problem	FM170
Myrsinaceae	*Maesa lanceoloata *Forssk.	Kaggano	T	Powdered fresh/dry root mixed with residue of local 'areqie' or 'tella' is given as drink	Anthrax	FM210
				Powdered fresh/dry root and *Vernonia amygdalina *leaf mixed with residue of local 'areqie' or 'tella' is given as drink	Blackleg	
Oleaceae	*Olea europaea *L.	Wayrro	T	The root powder is smoke in livestock fence	Mental problem	FM187
Papaveraceae	*Argemone mexicana *L.	Kossalae	H	Crushed and pounded fresh leaf mixed with roots of *Solanum indicum *in cold water is given as a drunk	Bloody Urine	FM81
			H	Powdered fresh leaf mixed with residue of local 'tella' or 'areqie' is given orally	Diarrhea	
			H	Crushed and pounded fresh leaf mixed with leaf of *Vernonia amygdalina *is given orally.	Intestinal parasites	
Polygonaceae	*Rumex nepalensis *Spreng.	Dangago	H	Powdered fresh/dry stem mixed with butter is applied topically	Wound	FM10
Rubiaceae	*Pentas schimperiana *(A. Rich) Vatke	Dibexxo	Sh	Root bark fine powder is mixed with water given orally	Mental problem	FM78
Santalaceae	*Osyris quadripartite *Decn.	Watto	Sh	Powdered fresh/dry fruit mixed with water is given orally for three days and applied topically on infected body part	Skin infection	FM105
Sapindaceae	*Dodonaea angustifolia *L.F.	Ittechhae	Sh	Crushed, pounded dry fruit with water is applied	Ectoparasite	FM83
				Powdered dry fruit with water is given orally	Lymphatic swelling	
Simaroubaceae	*Brucea antidysenterica *J.F.Mill	Kapparro	Sh	Powder of fresh/dry root bark is applied topically	Wound	FM202
Solanaceae	*Datura stramonium *L.	Atsefareceae	H	Crushed, pounded fresh/dry root mixed with *Parthenium hysterophorus *leaf applied topically	Wound	FM47
Solanaceae	*Discopodium penninervum*	Serbae	T	Rubbing affected part with fresh/dry crushed leaf	Inability to walk properly	FM198
Solanaceae	*Solanum indicum *L.	Dimoxxa embayo	Sh	A cup of fresh/dry root powder concocted with *Vernonia amygdalina *leaf with seven cups of water is boiled until only one cup of mixture remains then mixed with the residue of 'tella' and ' areqie' is given for as drink for three days.	Blackleg	FM104
				Crushed, pounded fresh/dry root and root of *Rhus vulgaris *mixed with water is given as drink for 2 to 3 days.	Anthrax	
				Concoction of crushed, pounded fresh/dry root with *Vernonia amygdalina *leaf is given as drink	Cough	
Tiliaceae	*Grewia ferruginea *Hochst ex A. Rich	Ogomdii	Sh	Crushed, pounded fresh/dry root bark with roots of *Ensete ventricosum *and mixed with water and kept overnight is given orally	Cough	FM121
Verbenaceae	*Lantana camara *L.	Yewof kollo	Sh	Dry stem powdered mixed with water is given orally	Diarrhea	FM146

**Table 5:** List of medicinal plants for treating both human and livestock diseases in the study area, Wonago Woreda

**Families**	**Scientific name**	**Local name**	**Habit**	**Preparation and application**	**Diseases treated**	**Voucher Number**
Acanthaceae	*Justicia schimperiana *(Hochst.ex A. Nees) T.Anders	Dummiuggae	Sh	Pounded fresh/dry leaves is concocted with bark of *Croton macrostachyus *is taken orally for three days.	Intestinal parasites	FM30
Asparagaceae	*Asparagus africanus *L.	'Uffae '	Sh	Powder of dry root with butter is applied on wound	Wound	FM206
Asteraceae	*Vernonia auriculifera *Hiern	Dangireto	Sh	Crushed, pounded and mix with cold water, one cup of the filtrate is given for adult, one-half of the cup for children for three days. For livestock Crushed, pounded root mixed with cold water is administered orally	Snake poison	FM144
Asteraceae	*Xantium strumarium *L.	Dehanekayae	H	The plant part squeezing it through clean locally made cloth for five days on affected part or wash the affected part for both diseases.	Skin infection	FM9
Boragnaceae	*Cordia africana *Lam.	Waddissa	T	Powdered dry root bark is sprinkled on burning charcoal and smoke is inhaled covered by cloth. For livestock root bark is smoked in the barn	Evil eye	FM167
Celastraceae	*Maytenus senegalensis *(Lam.) Excell	Shekko	Sh	Powdered fresh/dry seed with water or butter is taken with coffee or tea as drink for five days.	Epilepsy	FM54
				Powdered fresh/dry seed with *Ocimum lamiifolium *seed is take with coffee as drink	Headache	
				For livestock root powder mixed with leaf of *Ocimum lamiifolium *is administered orally	Febrile Disease	
Malvaceae	*Sida schimperiana *Hochst. ex A.Rich	Gebresede	Sh	Crushed, pounded, and boiled with water and cooled for 2 hours and 2 glasses are served as a drink.	Epilepsy	FM170
				For livestock leaf powder is mixed with water is administered orally for three days before grazing	Mental problem	
Polygonaceae	*Rumex nepalensis *Spreng.	Dangago	H	Paste of fresh/dry stem powder with butter is applied topically	Wound	FM10
Rubiaceae	*Pentas schimperiana *(A. Rich) Vatke	Dibexxo	Sh	Fresh/dry root bark powder mixed with water is taken orally	Epilepsy(human) Mental problem (livestock)	FM78
Sapindaceae	*Dodonaea angustifolia *L.F.	Ittechhae	Sh	Crushed, pounded dry fruit with water is applied	Ectoparasite	FM83
				Powdered dry fruit with water is given orally	Lymphatic swelling	
Simaroubaceae	*Brucea antidysenterica *J.F.Mill	Kapparro	Sh	Powdered fresh root bark mixed with water is applied topically	Wound	FM202
Tiliaceae	*Grewia ferruginea *Hochst ex A. Rich	Ogomdii	Sh	Crushed, pounded fresh/dry root bark with roots of *Ensete ventricosum *and mixed with water and kept overnight is given orally	Cough	FM121
Verbenaceae	*Lantana camara *L.	Yewef kollo	Sh	Dry stem powder mixed with water is taken orally	Diarrhea	FM146

The highest number of plant species was found in Asteraceae with 10 plant species followed by Solanaceae with 6 plant species, Euphorbiaceae and Fabaceae each with 5 plant species, Celastraceae and Cucurbitaceae with 3 plant species each (Table 3, 4, 5).

**Figure 3:**
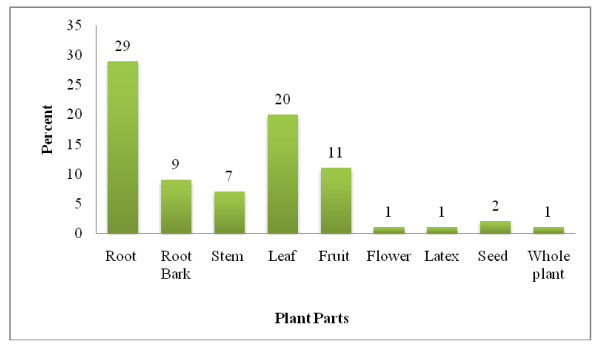
**Parts of medicinal plants used as remedy in the study area, Wonago Woreda**.

The shrubs were the most harvested for medicinal purpose and were represented with 35 (43.2%) plant species followed by 28(34.5%) herbs, 17 (20.9%) trees and 1(1.2%) climbers. The most commonly used plant parts for remedy preparations were roots (35.8%), followed by leaves (24.6%) (Fig. 3).

Remedies were mainly prepared in the form of powder, concoction and decoction (Table 6). Healers used various units of measurement such as fingered length (e.g. for root, root bark, and stem), pinch (e.g. for powdered plant parts) and numbers (e.g. for leaves, seeds, fruits and flowers) were used to estimate and fix the dosage of the medicine. The methods of administration of herbal medicines were 48(59.2%) internal, particularly oral, followed by 22(27.1%) dermal and 10(12.3%) nasal.

**Table 6:** Preparation methods of traditional medicine in the study area, Wonago Woreda

**Preparation methods**	**Preparations**	**Percent**
Powder	46	37.3
Crushing and pounding	42	34.1
Chewing	10	8.1
Concoction	7	5.6
Decoction	2	1.6
Others	6	13.0

#### Ranking of medicinal plants on their uses

Malaria and diarrhea were the most common diseases for which large number of patients visits the traditional medicinal practitioners. *Vernonia amygdalina *was the most preferred as effective treatment against malaria (Table 7) and *Croton macrostachyus *was preferred among the medicinal plants that were reported by more informants as a remedy to diarrhea (Table 8).

**Table 7:** Preference ranking of medicinal plants used for treating malaria in the study area, Wonago Woreda

**List of medicinal plants**	**R1**	**R2**	**R3**	**R4**	**R5**	**R6**	**R7**	**R8**	**Total**	**rank**
*Allium sativum*	3	2	5	3	3	2	3	3	24	3^rd^
*Lepidium sativum*	2	1	2	2	1	3	2	2	15	4^th^
*Croton macrostachyus*	4	5	3	4	4	5	5	4	34	2^nd^
*Phytoloca dodeccandra*	1	4	1	1	2	1	1	1	12	5^th^
*Vernonia amygdlania*	5	3	4	5	5	4	4	5	35	1^st^

**Table 8:** Preference ranking of medicinal plant species used to treat diarrhea in the study area, Wonago Woreda

**List of medicinal plants**	**R1**	**R2**	**R3**	**R4**	**R5**	**R6**	**R7**	**R8**	**Total**	**rank**
*Ensete ventricosum*	1	2	1	2	2	1	2	2	13	4th
*Vernonia amygdalina*	2	3	2	3	2	3	2	1	18	2^nd^
*Colocasia esculenta*	1	1	1	2	2	1	1	2	11	5^th^
*Croton macrostachyus*	4	3	3	2	3	2	1	3	21	1^st^
*Hagenia abyssinica*	2	1	3	1	1	3	3	2	16	3^rd^

#### Informant consensus factor (ICF)

Diseases that were found to be prevalent in the area were treated by variety of medicinal plants. The category: malaria, fever and headache have the highest 0.82 ICF followed by ascariasis and diarrhea, and intestinal parasite and stomachache each with 0.78 ICF (Table 9).

**Table 9:** Informant consensus factor by categories of diseases in the study area, Wonago Woreda

**Category**	**Species**	**(%) All Species**	**Use citations**	**(%) All use citations**	**ICF**
Malaria, Fever and headache	10	19%	52	39%	0.82
Ascariasis and diarrhea	11	20%	47	35%	0.78
Intestinal parasite and stomachache	5	9%	19	14%	0.78
Gonorrhea & sexual impotence in men	5	9%	16	12%	0.73
Abdominal pain and amoebas	6	11%	19	14%	0.72
Ring worm and wounds	7	13%	16	12%	0.60
Bronchitis and cough	6	11%	12	9%	0.55
Cancerous Swelling	5	9%	9	7%	0.50

#### Multiple uses of plants and effect on the conservation of the medicinal plants

The people in the Woreda relied on naturally growing plant species for various purposes such as construction, firewood, washing, cash income and charcoal. *Croton macrostachyus *was used for variety of services by the community followed by *Millettia ferruginea*; however, each plant species was used for a given specific service such as *Phytolacca dodecandra *was used for washing more often than the other plants (Table 10).

**Table 10:** Direct matrix ranking of medicinal plants with different uses other than medicinal value (total score of ten informants) in the study area, Wonago Woreda

**Uses**	***Croton macrostachyus***	***Phytolacca dodecandra***	***Coffea arabica***	***Cordia africana***	***Millettia ferruginea***
Construction	31	9	26	24	23
Cash income	29	12	27	13	19
Washing	21	26	0	19	29
Firewood	13	16	23	22	19
Charcoal	18	7	19	11	15
Total	112	70	95	89	105
Rank	1st	5th	3rd	4th	2nd

The medicinal plants in Wonago Woreda were threatened by natural and human made factors. Agricultural expansion was found to be the main threat followed by fire wood collection (Table 11).

**Table 11:** Priority ranking of factors perceived as threats to medicinal plants based on their level of destructive effects in the study area, Wonago Woreda (values 1-6 were given: 1 is the least destructive threat and 6 is the most destructive threat)

	**Respondents (R1-R6)**						**Total**	**Percent**	**Rank**
				
**Factors**	**R1**	**R2**	**R3**	**R4**	**R5**	**R6**			
Drought	3	4	2	3	6	3	21	16.5	4^th^
Grazing	5	1	3	5	4	5	23	18.1	3^rd^
Urbanization	1	5	4	1	3	1	15	11.8	5^th^
Agricultural expansion	6	2	6	6	5	6	31	24.4	1^st^
Fire wood	4	6	5	4	1	4	24	18.8	2^nd^
Construction	2	3	1	2	2	3	13	10.2	6^th^

## Discussion

### Distribution of medicinal plants in the study area

Most of the shrubs were collected from woodlands, rocky surfaces, secondary forests and home gardens. The herbs were mostly found in woodland, grazing land and farmlands. The tree species were found in open woodland, farm borders, roadsides, live fences and in coffee plantation areas. Medicinal plants like *Allium sativum*, *Artemisia abyssinica*, *Capsicum anuum*, *Lepidium sativum*, *Ensete ventricosum*, *Nicotiana tabacum*, *Ocimum lamiifolium*, *Ruta chalepensis*, and *Zingiber officinale *were restricted to farmlands, farm boarders, live fences and home gardens. Hunde [18], Mohammed [19], Tollosa [20] and Awas and Asfaw [21] used similar approaches to identify sites of collection of medicinal plants.

### Natural vegetation and home garden diversity

In this study, the number of medicinal plants collected from the natural vegetation is more than home gardens. This is also true to the studies conducted in different parts of the country. 90.43% of medicinal plants in Mana Angetu District, southeastern Ethiopia [22]; 92% of medicinal plants around 'Dheeraa' town, Arsi Zone, Ethiopia [23]; 71% of the medicinal plants of the 'Berta' people in western Ethiopia [24] and 85.71% of medicinal plants of Sekoru District, Jimma Zone, Southwestern Ethiopia [25] are obtained from the natural vegetation. Asfaw [26] reported that only 6% of the plants maintained in home gardens in Ethiopia are primarily cultivated for their medicinal value. Some of the medicinal plants cultivated provided a number of services to the local people because the primary function of these home gardens was to produce foodstuffs. This might be because of high population density and shortage of land for cultivation in the area [27].

### Medicinal plants

The medicinal plant species recorded in Wonago are also used as remedies in other parts of Ethiopia and Africa. Among the total of Seventy-two medicinal plant species investigated in this study, 22 species are mentioned in Taddese [28]; 20 species in Wondimu *et al*. [23]; 11 species in Taddese and Demissew [29]; 23 species in Tamene [30]; 21 species in Hunde [18]; 11 species in Balemie *et al*. [31]; 39 species in Lulekal *et al*. [22]; 21 species in Teklehaymanot and Giday [32] and 17 species in Teklehaymanot *et al*. [33]. In Africa, 13 medicinal plant species are documented by Anokbongo [34] and 16 by Iwn [35].

Some of the medicinal plants in this study were used to treat specific diseases:*Vernonia amygdalina *Del., *Momordica foetida *Schumach, *Ocimum lamiifolium *Hochst. Ex Benth., and *Lantana camara *L. are used as treatment for malaria and associated illness in Budiope county Uganda [36]. *Croton macrostachyus *Del., *Datura stramonium *L., *Eucalyptus globules *Labill, *Euphorbia candelabrum *Kostshy, *Euphorbia tirucalli *L., *Prunus africana *(Hook.F.) Kalkam, *and Ricinus communis *L. in Central Kenya [37], and *Calpurnia aurea *(Alt.) Benth. *and Phytolacca dodecandra *L'Herit in Ethiopia [38] are used for treatment of skin disorders.

*Allium sativum *L., *Lagenaria siceraria *(Molina) Standl., *Zingiber officinale *Rosc., *Capsicum annuum *L, and *Ricinus communis *L. are used as anthelmintics in traditional veterinary practices in Sahiwal district of Punjab, Pakistan; and the anthelmintic activity of the first three medicinal plants is scientifically validated through *in vitro *and *in vivo *tests [39].

The medicinal plants that were presumed to be effective in treating a certain disease had higher ICF values, which indicated that these diseases were more common than those with low ICF: malaria and headache (82.3%), ascariasis and diarrhea (78.2%), and intestinal parasite and stomachache (77.7%).

The most widely used plant remedies by people of Wonago were obtained from shrubs (43.2%) followed by herbs (34.5%). The documented data showed that the majority of medicinal plants from natural vegetation were shrubs and herbs; they were relatively common in the study area compared to medicinal tree species. This finding agrees with the findings of Tamene [30], Hunde [18] Yineger and Yewhalaw [25], Giday and Amani [40] and Lulekal *et al*. [22]. However, the finding of Birhanu [41]; Mohammed [19]; Gebre [42] and Teklehaymanot and Giday [32] shows that herbs are the primary habit form.

The most widely sought plant parts in the preparation of remedies were the root [22], root bark, leaves and stems. The popularity of these parts has serious consequences from both ecological point of view and from the survival of the medicinal plant species [41]. Tesfu *et al*. (Tesfu CB, Mengistu B, W/Aregay G: Women lead in protecting food germplasm and herbs for health in Ethiopia, Submitted) reported that some plant species such as *Dracaena steudneri*, *Hagenia abyssinica *and *Securidaca longepedunculata *that are harvested for their roots, barks or whole plants in many parts of Ethiopia have become scarce and so difficult to find. On the other hand, collecting leaves alone could not pose a lasting danger to the continuity of an individual plant compared with the collection of roots, bark, stem or whole plant.

The route of application, oral (42%), is popular as in the finding of Abebe and Ayehu [43] who reported as the leading route of application used in northern Ethiopia. It is also in agreement with the result of various ethnobotanical studies conducted elsewhere in Ethiopia [18,21,22,31,40,41,44,45] and indicates oral as the predominant route of application.

The informants' responses indicated that there were variations in dosages of remedies, unit of measurement of remedies, duration and time that were prescribed for the same kind of health problems. The major factors that determine the amount to be given were age, physical fitness, stage of illness, pregnancy and presence or absence of any disease other than the disease to be treated. Getahun [46], Sofowara [47] and Abebe [2] have also discussed lack of precision and standardization as a drawback of the traditional health care system.

### Conservation and threats of medicinal plants

Some traditional practitioners had started to conserve medicinal plants by growing them in home gardens. Such as *Ruta chalepensis*, *Rhus vulgaris*, *Ocimum lamiifolium*, *Artemisia abyssinica *and *Artemisia afra *similar to the observation made by Kansheiae [27]. In most scenarios, the home gardens are fenced and protect the medicinal plants from grazing and unwise harvesting [48].

The main threat for medicinal plants in the natural vegetation was agricultural expansion (24.4%). Most of the respondents perceived urbanization and construction as the least destructive factors contributing to 11.8% and 10.2% of the total score, respectively. The rise in *Coffea arabica *and *Catha edulis *price on the market were some of the contributing factors for the expansion of agriculture. The other factor was the number of young farmers who were anxious to have their own agricultural land; hence, clearing of natural vegetation and expanding agricultural land was almost a daily activity in the study area. Nevertheless, during the field study, it was observed that large number of big trees of *Macaranga capensis, Olea europaea, Pouteria adolfi-friederici*, and *Syzygium guineense *were removed by the local people to prepare the forestlands for agricultural purpose. These factors combined with the natural vulnerability of the area may lead to further reduction in natural habitats of the medicinal plants. Pressure from agricultural expansion, wide spread cutting for fuel wood combined with seasonal drought is also reported in Balemie *et al *[31], Lulekal *et al*. [22], Nanyingi *et al*., [48], Kelbessa *et al*. [49] and Yineger *et al*. [50] as main factor for environmental degradation.

The conservation of medical plants in the study area was limited except in *Juniperous- Eucalyptus *dominated plantation, which was the only protected natural vegetation areas. Rather, the peoples' culture and spiritual beliefs somehow had helped in the conservation of medicinal plants. For instance, the claim of the traditional healers that medicinal plants will be effective only if cut and administered by the healers or healers' reletives had helped in the conservation of the medicinal plants. Also, the collection of medicinal plants in specific season, for example, at the end of the Ethiopian calendar year in 'Pagume' enabled the plants to regenerate and complete their life cycle. This is true mostly for annuals, those whose leaves, fruits and seeds are used, if other destructive pressures are kept at low level.

## Conclusion

Traditional medicinal plants were harvested mostly from natural vegetation area followed by home gardens. They were also obtained from roadsides, farmlands and live fences. The medicinal plants in the natural vegetation were under threat and to tackle these problems traditional healers had turned their face towards home gardens. However, traditional healers still depend largely on naturally growing species because of their belief that those species in the natural vegetation are more effective in the prevention and treatment of diseases and health problems. Furthermore, the documented medicinal plants can be used as a basis for further studies on the regions medicinal plants knowledge and for future phytochemical and pharmacological studies.

## Declaration of competing interests

The authors declare that they have no competing interests.

## Authors' contributions

The authors have made substantive intellectual contributions to this study in data collection, identification of plants, preparation of the manuscript and proof reading.

## Supplementary Material

Additional file 1 **List of plant species collected from natural vegetation in the study area**. It shows plants collected from the natural vegetation and those that are used as medicine in the community. Click here for file
