# Supplementary material for: An ethnobotanical study of medicinal plants in Wonago Woreda, SNNPR, Ethiopia
Source: J Ethnobiol Ethnomed. 2009 Oct 12;5:28. doi: 10.1186/1746-4269-5-28 (PMC2769162; doi:10.1186/1746-4269-5-28)
Supplement: Additional file 1 — List of plant species collected from natural vegetation in the study area. It shows plants collected from the natural vegetation and those that are used as medicine in the community. [file 1746-4269-5-28-S1.pdf]

Additional file

Additional file 1:List of plant species collected from natural vegetation in the study area, Wonago Woreda (Habit: T-tree, Sh-shrub, H-herb, Cl-climber, and Ep - epiphytes, (M) – Medicinal Plant)

Families	Scientific name	Local name	Habit	Voucher No.
Acanthaceae	*Acanthus polystachius* Delile	Dedexxo	Sh	FM97
Acanthaceae	*Acanthus eminens* C.B.Clarke	Comexxo	Sh	FM201
Acanthaceae	*Justicia schimperiana* (Hochst. ex A. Nees) T. Anders	Dummiuggae	Sh(M)	FM30
Amaranthaceae	*Cyathula uncinulata* (Schrad.) Schinz.	Gixxaa	H	FM4
Amaranthaceae	*Cyathula cylindrica* Maq.	Gixxaa	H	FM80
Amaranthaceae	*Achyranthes aspera* L.	Derrgu	H(M)	FM115
Amaranthaceae	*Amaranthus spinosus* L.	Alemae	H(M)	FM180
Anacardiaceae	*Rhus vulgaris* Meikle	Xxugutae	Sh(M)	FM57
Anacardiaceae	*Schinus molle* L.		T	FM69
Apiaceae	*Oenanthe palustris* (Chiov.) Norman		H	FM91
Apiaceae	*Centella asiatica* (L.) Urban.	Xxerexxo	H	FM125
Apiaceae	*Foeniculum vulgare* Mill.	Mello	H(M)	FM193
Apocynaceae	*Maytenus arbutifolia* (A. Rich) Wilczek	Kombollechae	Sh(M)	FM138
Araceae	*Arisaema ennephyllum* Hochst. ex A. Rich	Badenxxo	T	FM99
Araliaceae	*Polyscias fulva* (Hiern) Harms	Teleha	T	FM179
Arecaceae	*Phoenix reclinata* Jacq.	Xenebo	T	FM106
Asclepiadaceae	*Gomphocarpus purpurascens* A. Rich	Mexxino	Sh(M)	FM142

Asclepiadaceae	*Kanahla laniflora* (Forssk.) R. Br.	Wundiffo	Sh(M)	FM136
Asparagaceae	*Asparagus africanus* Lam.	Uffae	Sh(M)	FM206
Asteraceae	*Gnaphalium rubriforum* Hilliard	Nophixxo	H	FM12
Asteraceae	*Cirsium englerianum* O. Hoffm.	Galigloo	H	FM64
Asteraceae	*Sphaeranthus suaveolens* (Forssk.) DC		H	FM77
Asteraceae	*Xanthium spinosum* L.		H	FM88
Asteraceae	*Tagetes minuta* L.	Chebbo	H	FM148
Asteraceae	*Dicrocephala integrifolia* (L.F..) O. Kuntze	Gishtu	H	FM151
Asteraceae	*Echinops amplexicaulis* Oliv.	Messichae	H	FM173
Asteraceae	*Crepis rueppellii* Sch. Bip.		H	FM178
Asteraceae	*Guizotia scabra* (Vis.) Chiov.		H	FM207
Asteraceae	*Parthenium hysterophorus* L.	Partinumae	H(M)	FM2
Asteraceae	*Xanthium strumarium* L.	Dehanekayae	H(M)	FM9
Asteraceae	*Carduus leptacanthus* Fresen.	Guccino	H(M)	FM86
Asteraceae	*Cirsium englerianum* O. Hoffm.	Galigloo	H(M)	FM143
Asteraceae	*Guizotia abyssinica* (L.F.) Cass.	Mechae	H(M)	FM154
Asteraceae	*Laggera alata* (D.Don) Sch. Big. ex Oliv.	Luggae	Sh	FM112
Asteraceae	*Laggera crispata* (Vahl) Hepper	Hopphicho	Sh	FM116
Asteraceae	*Vernonia amygdalina* Del.	Ebicha	Sh(M)	FM31
Asteraceae	*Vernonia auriculifera* Hiern.	Dangireto	Sh(M)	FM144
Bignonaceae	*Jacaranda mimosifolia* D. Don.	Y/zafae	T	FM145

Boraginaceae	*Cynoglossum coervleum* Hochst ex A. Rich	Korchibae	H	FM135
Boraginaceae	*Cynoglossum lanceolatum* Forsk.	Korchibae	H(M)	FM114
Boraginaceae	*Cordia africana* Lam.	Waddissa	T	FM167
Burseraceae	*Boswellia neglecta* S. Moore	Galgalchae	T	FM175
Caryophyllaceae	*Drymaria cordata* (L.) Schltes		H	FM166
Caryophyllaceae	*Stellaria sennii* Chiov.		H(M)	FM188
Casuarinaceae	*Casuarina cunninghamiana* Miq.	Shewshewae	T(M)	FM76
Celastraceae	*Catha edulis* (Vahl.) Forssk ex Endl.	Chatae	Sh(M)	FM19
Celastraceae	*Maytenus senegalensis* (L.) Excell	Shekko	Sh(M)	FM54
Celastraceae	*Maytenus arbutifolia* (A. Rich) Wilczek	Kombollechae	Sh	FM138
Clustiaceae	*Hypericum peplidifolium* A.Rich	Dammae	Sh	FM195
Clustiaceae	*Hypericum revolutum* Vahl.		Sh(M)	FM93
Commelinaceae	*Commelina diffusa* Burm. F.	W/hankurae	H	FM129
Cucurbitaceae	*Dactyliandra stenfaninii* (Chiov.) C. Jeffrey		Cl	FM192
Cucurbitaceae	*Lagenaria siceraria* (Molina) Standl.	Botto	H(M)	FM205
Cucurbitaceae	*Momordica foetida Schumach* &Thonn.	Yubarrae	Sh(M)	FM108
Cuperessaceae	*Juniperus procera* Hochst ex Engl.	Xxdiae	T	FM159
Cyperaceae	*Cyperus mundfii* (Nees) Kunth	Rogrogal	H	FM82
Cyperaceae	*Cyperus dichroostachyus* A. Rich	Embuteteya	H	FM147
Dracaenaceae	*Dracaena afromontana* Mild Br.	Woreko	T	FM174
Dracaenaceae	*Dracaena steudneri* Engl.	Afrafartu	T(M)	FM37

Euphorbiaceae	*Manihot esculenta* Granz	Yammenoi	H	FM119
Euphorbiaceae	*Tragia cinerea* (Pax) Gilbert & Radcl. Smith.	Aleblabitae	H(M)	FM87
Euphorbiaceae	*Euphorbia pulcherrina* (R. Grah.) Willd	Abababo	Sh	FM41
Euphorbiaceae	*Euphorbia tirucalli* L.	Kinchbae	Sh(M)	FM40
Euphorbiaceae	*Euphorbia candelabrum* Kostshy	Addama	Sh(M)	FM48
Euphorbiaceae	Ricinus communis L.	Gulloo	Sh(M)	FM71
Euphorbiaceae	*Macaranga capensis* (Baill.) Sim	Yunddae	T	FM32
Euphorbiaceae	*Sapium ellipticum* (Krauss) Pax	Waggiso	T	FM211
Euphorbiaceae	*Croton macrostachyus* Del.	Bissano	T(M)	FM162
Fabaceae	*Caesalpinia decapetala* (Roth) Alston	Konnxxera	Cl	FM186
Fabaceae	*Crotalaria pallida* Ait.		H	FM95
Fabaceae	*Senna occidentalis* (L.) Link	Assenmeka	H(M)	FM103
Fabaceae	*Indigofera emarginella* A. Rich	Boffeae	H(M)	FM172
Fabaceae	*Sesbania sesban* (L.) Merr.		Sh	FM113
Fabaceae	*Leucaena leucocephala* L.		Sh	FM169
Fabaceae	*Calpurnia aurea* (Alt.) Benth.	Chekketa	Sh(M)	FM98
Fabaceae	*Acacia abyssinica* Hochst. ex Benth.	wochhoo	T	FM100
Fabaceae	*Delonix regia* (Boj. ex Hook.) Ref.	Yed/zafae	T	FM130
Fabaceae	*Erythrina brucei* Schweinf.	Korchae	T(M)	FM79
Fabaceae	*Millettia ferruginea* (Hochst.) Bak.	Berberae	T(M)	FM190
Fabaceae	*Pisium sativum* L.	Attaro	H	FM128
Lamiaceae	*Saliva nilotica* Juss. ex Jacq.		H	FM85

Lamiaceae	*Satureja paradoxa* (Vatke) Engler	Naddae	H	FM204
Lamiaceae	*Otostegia tomentosa* A. Rich.	Mukalonii	Sh	FM63
Lamiaceae	*Pyconostachys eminii* Gurke	Shegino	Sh	FM102
Lauraceae	*Persea americana* Mill.	Kokae	T	FM75
Loganiaceae	*Buddleja polystachya* Fresen.	Affarao	Sh(M)	FM7
Loranthaceae	*Englerina woodfordioides* (Schweinf.) M.Gilbert	Eritobekkesisa	EP	FM141
Malvaceae	*Hibiscus flavifolius* Ulbar.	Bayirro	H	FM109
Malvaceae	*Malva verticillata* L.	Xxummo	H	FM160
Malvaceae	*Sida schimperiana* Hochst. ex A. Rich.	Gebresede	Sh(M)	FM170
Melastomataceae	*Dissotis senegambiensis* (Guill. & Pern) Triana	Arkaae	H	FM156
Meliaceae	*Melia azedarach* L.	Nemae	T	FM118
Meliaceae	*Ekebergia capensis* Sparm.	Sessa	T	FM185
Meliaceae	*Trichilia dregeana* Sond.	Yumbarro	T(M)	FM126
Melianthaceae	*Bersama abyssinica* Fresen.	Jejjeba	Sh(M)	FM163
Menispermaceae	*Stephania abyssinica* (Dilloy and A. Rich) Walp.	Shesheno	H	FM101
Moraceae	*Ficus sur Forssk*.	Sholae	T	FM161
Moraceae	*Ficus ovata* Vahl	Shollae	T(M)	FM153
Moringaceae	*Moringa stenopetala* L.	Shefferaw	T(M)	FM62
Myrsinaceae	*Embelia schimperi* Vatke.	Sharrengo	Sh(M)	FM122
Myrsinaceae	*Maesa lanceolata* Forssk.	Kaggano	T(M)	FM210

Myrtaceae	*Psidium guajava* L.	Gettamae	Sh	FM89
Myrtaceae	*Callistemon citrinus* (Curtis) Skeels	Paricho	Sh	FM155
Myrtaceae	*Syzygium guineense* (Willd.) DC.	Debobessa	T	FM117
Myrtaceae	*Eucalyptus saligna* Smith	K/barzafae	T	FM157
Myrtaceae	*Eucalyptus globulus* Labill	D/barzafae	T(M)	FM150
Oleaceae	Jasminum abyssinicum Hochst. ex A. Rich.	Tembele	Cl	FM177
Oleaceae	*Olea europaea* L.	Wayrro	T(M)	FM187
Oxalidaceae	*Oxalis radicosa* A. Rich.		H	FM127
Papaveraceae	*Argemone mexicana* L.	Kossalae	H(M)	FM81
Passifloraceae	*Passiflora edulis* Sims	Woyane	Cl	FM134
Phytolaccaceae	*Phytolacca dodecandra* L'Herit.	Indodae	Sh(M)	FM176
Pinaceae	*Pinus radiata* L.		T	FM92
Plantaginaceae	*Plantago lanceolata* L.	Diggixxae	H	FM107
Poaceae	*Cynodon dactylon* (L.) Pers.	Serdoo	H	FM110
Poaceae	*Hyparrhenia filipendula* (Hochst.) Stat	Gedecho	H	FM137
Poaceae	*Panicum maximum* Jacq.		H	FM140
Poaceae	*Arundinaria alpine* K.Schum.	Kerrkeha	H	FM152
Poaceae	*Snowdenia polystachya* (Fresen.) Pilg.		H	FM183
Poaceae	Arundo donax L.	Serrdo	H	FM214
Podocarpaceae	*Podocarpus falcatus* (Thunb.) Mirb.	Zigebo	T(M)	FM11
Polygonaceae	*Rumex nepalensis* Spreng.	Dangago	H(M)	FM10
Protaceae	*Grevillea robusta* R. Br.		T	FM182

Resedaceae	*Caylusea abyssinica* (Fresen.) Fisch. & Mey.	Sheggitae	H(M)	FM131
Rhamnaceae	*Rhamnus prinoides* L'Herit.	Gesho	Sh	FM49
Rosaceae	*Alchemilla cryptantha* A. Rich	Imbricho	H	FM124
Rosaceae	*Rubus steudneri* Shweinf.	Engoraae	Sh	FM74
Rosaceae	*Rubus apetalus* Poir.	Engorae	Sh	FM149
Rosaceae	*Malus sylvestris* Miller	Apiliae	T	FM53
Rosaceae	*Hagenia abyssinica* (Brucie.) J. F. Gmel	Kossae	T(M)	FM120
Rosaceae	*Prunus africana* (Hook. F.) Kalkam	T/kaka	T(M)	FM209
Rubiaceae	*Coffea arabica* L.	Bunno	Sh(M)	FM1
Rubiaceae	*Pentas schimperiana* (A. Rich) Vatke	Dibexxo	Sh(M)	FM78
Rutaceae	*Vepris dainellii* (Pichi-Serm.) Kokwaro		Sh	FM133
Rutaceae	*Citrus medica* L.	Burtukanae	Sh	FM189
Rutaceae	*Citrus limon* (L.) Burm.F.	Lomae	Sh(M)	FM123
Santalaceae	*Osyris quadripartita* Decn.	Watto	Sh(M)	FM105
Sapindaceae	*Dodonaea angustifolia* L.	Ittechhae	Sh	FM83
Sapindaceae	*Allophylus abyssinicus* (Hochst.) Radlk.	Embesae	T	FM132
Sapotaceae	*Pouteria adolfi-friederici* (Engl.) Baenni	Quarero	T	FM200
Simaroubaceae	*Brucea antidysenterica* J.F. Mill.	Kapparro	Sh(M)	FM202
Solanaceae	*Datura stramonium* L.	Ashefareceae	H(M)	FM47
Solanaceae	*Solanum americanum* Miller	Dinicha	Sh	FM73
Solanaceae	*Solanum anguiniri* Lam.	Embayo	Sh	FM203
Solanaceae	*Nicotiana tabacum* L.	Tambo	Sh(M)	FM56

Solanaceae	*Solanum indicum* L.	Dimoxxa embayo	Sh(M)	FM104
Solanaceae	*Discopodium penninervum* Hochst.	Serbae	T(M)	FM198
Thelypteridaceae	*Thelypteris confluens* (Thunb.) Morton.		H	FM8
Tiliaceae	*Grewia ferruginea* Hochst.ex A. Rich.	Ogomdii	Sh(M)	FM121
Tiliaceae	*Triumfetta tomentosa* Boj.	Kombocho	Sh(M)	FM171
Urticaceae	*Droguetia iners* (Forssk.) Schweinf.	Haroxxae	H	FM191
Urticaceae	*Girardinia diversifolia* (Link.)Friis	Mutate	H	FM199
Verbenaceae	*Lippia adoensis* Hochst ex Walp.	Kessae	Sh	FM197
Verbenaceae	*Lantana camara* L.	Yewoffekolo	Sh(M)	FM146
Vitaceae	*Cissus qudriangularis* L.	Chobihada	Cl	FM27
